# Parliament and the Professions

**Published:** 1921-08-13

**Authors:** 


### Parliament and the Professions.

### Administrative Service Doctors.

By the War Secretary and the Parliamentary Secretary to the Admiralty Commander Bellairs was informed that in the Army and Navy respectively there are 166 and 8 medical officers whose work is of an administrative or clerical nature only. In addition, in the Army there are 26 officers doing duty in territorial schools of instruction and in administrative medical posts with territorial divisions.

### Women Panel Doctors.

The Minister of Health stated that there are 117 women physicians at present acting as panel doctors in England and Wales, and the number of patients on their lists is 40,686.

### Regional Medical Officers.

In answer to a question put by Sir J. Harmood-Banner, the Minister of Health stated that thirty-three regional medical officers have been appointed by the Ministry, of whom one has died, and the vacancy is not filled. These officers act as medical referees on questions of incapacity of insured persons for work referred to them by the approved societies or raised direct by insured persons. In addition, they act as consultants to Insurance practitioners, and generally assist in maintaining the efficiency of the Insurance medical service.

### Insanity of Prisoners.

The Home Secretary explained to Mr. Lawson the procedure adopted when prison inmates become insane. These persons are certified under the Criminal Lunatics Act, 1884, by two members of the Prison Visiting Committee (who, of course, are Justices), and two legally qualified medical practitioners. If the prisoner is then removed to an asylum he continues to be subject to the Act until the expiration of his sentence, when he comes, if still insane, under the ordinary lunacy law.

### Tuberculosis Hospital, Boston.

Sir Alfred Mond stated that the Ministry of Health had withdrawn its offer for Norton House, Boston, which it was proposed should be used as a tuberculosis hospital, because of the suspension of various public health schemes on the instruction of the Government as to economy in public expenditure.

### Asylum Administration.

Dr. McDonald asked the Minister of Health a question on the recent book of Dr. Montagu Lomax, alleging inhumanity in the administration of asylums. Sir A. Mond said that the Visiting Committee of the asylum at which Dr. Lomax was employed as *locum tenens*, and to which he evidently refers, have been asked by the Board of Control for their observations. When these are received consideration will be given as to what further action is necessary.

### Staffs of Pensions Hospitals.

The following figures in reference to the staffs and patients of Ministry of Pensions Hospitals were given in answer to a question:

| | Staff | In-Patients | Out-Patients |
|---|---|---|---|
| Shepherd's Bush ... | 442 [265] | 543 | 511 |
| Netley ... ... | 109 [ 61] | 191 | 90 |
| Orpington ... ... | 458 [362] | 923 | 2 |
| Ruskin Park ... | 247 [136] | 327 | 436 |
| Bellahouston ... | 344 [177] | 717 | 1,168 |

The figures in brackets indicate the number of domestic and lay assistants included in the staff.

It was stated that the proportion of staff to patients varies with the type of case treated, and with the structural arrangements of the hospital.

